# Antiobesity and Antidiabetic Effects of *Portulaca oleracea* Powder Intake in High-Fat Diet-Induced Obese C57BL/6 Mice

**DOI:** 10.1155/2021/5587848

**Published:** 2021-06-25

**Authors:** Jae Hyun Jung, Su Bin Hwang, Hyeon Ju Park, Guang-Ri Jin, Bog Hieu Lee

**Affiliations:** Department of Food and Nutrition, Chung-Ang University, Gyeonggi-do 17546, Republic of Korea

## Abstract

This study investigated the hypothesis that *Portulaca oleracea* L. exerts antiobesity and antidiabetic effects by evaluating blood lipid profiles, blood glucose control factors, protein expression of lipid metabolism, and insulin sensitivity improvement. Three groups of high-fat diet (HFD) induced obese C57BL/6 mice (*n* = 8) received treatment with low (5%; HFD + PO5%) or high (10%; HFD + PO10%) concentrations of *P. oleracea* powder for 12 weeks or no treatment (HFD) and were compared with each other and a fourth control group. Weight gain was reduced by 34% in the HFD + PO10% group compared to the HFD group. Moreover, the perirenal and epididymal fat contents in the HFD + PO10% group were 6.3-fold and 1.5-fold, respectively, lower than those in the HFD group. The atherogenic index (AI) and cardiac risk factor (CRF) results in the *P. oleracea*-treated groups were significantly lower than those in the HFD group. The homeostasis model assessment of insulin resistance (HOMA-IR) levels was lower in the HFD + PO10% group than in the HFD group. The protein expression levels of the proliferator-activated receptor (PPAR)-*α*, glucose transporter (GLUT) 4 and PPAR-*γ* were upregulated in the HFD + PO10% group compared to the HFD group. However, the protein expression levels of tumor necrosis factor (TNF)-*α* were lower in the *P. oleracea*-treated groups than in the HFD group. Our results demonstrate that *P. oleracea* powder could be effectively used to treat and prevent obesity and diabetes-associated diseases through suppression of weight gain and reduction in body fat and blood glucose levels.

## 1. Introduction

The prevalence of chronic diseases such as obesity, type 2 diabetes, and cardiovascular disease has been steadily rising over the past several decades due to the westernization of eating habits, which involve a high intake of high-fat foods and red meat [1]. These changes are increasingly affecting the world population; therefore, the prevalence of dyslipidemia, arteriosclerosis, and cardiovascular disease is growing [2, 3]. The prevalence of obesity in Korean males has increased from 25.7% (phase I) to 37.9% (phase IV), according to the Korea National Health and Nutrition Examination Survey (KNHANES) [4]. Diabetes is becoming one of the most common health problems in developed countries such as Japan and North America and Europe [5]. In Korea, this disease will affect an estimated 4.3 million people by 2030 [6].

To help control obesity and maintain a normal weight, many studies have focused on appetite control, inhibition of lipid digestion and absorption, acceleration of energy expenditure through adaptive thermogenesis, and modulation of lipid metabolism [7]. Additionally, lipogenesis and lipolysis are important biochemical mechanisms that regulate lipid metabolism [7]. Peroxisome proliferator-activated receptor (PPAR)-*α*, which is associated with lipolysis, is abundantly expressed in brown fat and in the liver. These receptors mainly inhibit the growth and differentiation of adipocytes and promote lipolysis to regulate lipoprotein synthesis and tissue inflammatory responses. PPAR-*α* is also known to stimulate carnitine palmitoyltransferase (CPT)-1, which facilitates the transport of free fatty acids (FFAs) to mitochondria, thereby promoting *β*-oxidation of FFAs [8].

Adiponectin, the expression of which is regulated by factors such as PPAR-*γ*, reduces body weight and blood glucose while enhancing insulin sensitivity [9]. PPAR-*γ* is known to be a major regulator of adiponectin gene transcription and an important biological indicator associated with the insulin signaling pathway, insulin resistance, and inflammation [9, 10]. In contrast, tumor necrosis factor (TNF)-*α* has been found to be upregulated in obese patients and those suffering from diabetes and is known to directly contribute to reduced adiponectin expression [10]. Glucose transporter (GLUT) 4 upregulation also ameliorates insulin resistance [11].

*Portulaca oleracea* L. is an annual plant belonging to the Portulacaceae family and is rich in dopamine, L-noradrenaline, flavonoids, organic acids, coumarin (a type of polyphenol), vitamins (B1 and B2), and *γ*-linolenic acid, which is an *ω*-6 fatty acid [12–14]. *P. oleracea* has traditionally been used as a remedy for burns, headaches, cough, dyspnea, and arthritis and has been reported to possess several therapeutic properties such as antiulcer, anti-inflammatory, antidiabetic, antioxidant, and antibacterial effects [15, 16]. Although some studies have been conducted to assess the antiobesity and antidiabetic properties of *P. oleracea* [17–19], few studies have characterized the mechanisms that underlie this the therapeutic effects of this plant. Therefore, our study analyzed the effects of *P. oleracea* on several weight control-associated endpoints including blood glucose reduction and blood lipid profiles. High-fat diet-induced obese C57BL/6 mice (*n* = 8) were fed low (5%; HFD + PO5%) or high (10%; HFD + PO10%) concentrations of *P. oleracea* powder (*n* = 8) for 12 weeks, after which the mechanism of *P. oleracea* activity was investigated by characterizing the expression of related genes and proteins.

## 2. Materials and Methods

### 2.1. Diet Preparation and Formula Composition

The mice obtained for this study were divided into four groups: the control (CON) group, which was fed with normal mouse chow diet, the high-fat diet (HFD) group, the HFD + low (5%) *P. oleracea *powder (HFD + PO5%), and the HFD + high (10%) *P. oleracea* powder (HFD + PO10%) group. The CON group feed (NIH-41) and HFD group feed (TD.06414) were purchased from Dae Han Bio Link Co. Ltd. (Eumsung, Chungbuk, Korea).  Table 1 shows the composition of the experimental diet formula. The composition of *P. oleracea* powder purchased from Hanbit Farm (Yeongdeok, 73 Kyeongbuk, Korea) was indicated in Table 1.

### 2.2. Animal Breeding and Experimental Design

Male C57BL/6 mice were purchased from Dae Han Bio Link Co. Ltd. (Eumsung, Chungbuk, Korea). Thirty-two 5-week-old male C57BL/6 mice were employed in this study. Feeding environments were maintained at 23 ± 2°C, with a relative humidity of 50 ± 5%, and a 12 : 12 h light-dark cycle. All animal procedures were performed according to the Care and Use of Laboratory Animals guidelines of the National Institutes of Health and the Animal Welfare Act guidelines. The mice were allowed to acclimate to the laboratory environment for one week and provided free access to pelleted food and water. After the acclimation period, the mice were randomly assigned to the four experimental groups (*n* = 8/group) and raised for 12 weeks. Food intake and body weight were measured weekly and twice per week, respectively. The study was approved by the Institutional Animal Care and Use Committee (IACUC) from Chung-Ang University (approval ID: 2019-00006).

### 2.3. Blood Glucose Measurement

Blood glucose was measured seven times at 0, 2, 4, 6, 8, 10, and 12 weeks after the start of the feeding trials; this procedure was always conducted at the same time of day. Blood was collected from the tail vein, and blood glucose was measured using a blood glucose meter (Accu-Check Performa, Roche Diagnostics, Mannheim, Germany).

### 2.4. Intraperitoneal Glucose Tolerance Test

After a 12 h fast, the mice were injected with 10 *μ*l/g body weight (BW) of D-glucose (100 mg/ml in saline). Blood was collected from the tail vein at 0, 30, 60, 90, and 120 min after injection, and blood glucose was measured using a blood glucose meter (Accu-Check Performa, Roche Diagnostics, Mannheim, Germany).

### 2.5. Blood and Organ Collection

The mice were euthanized with CO_2_ gas after fasting for 12 h. Blood was collected via cardiac puncture and allowed to sit at room temperature for 30 min, followed by centrifuging at 3000 rpm for 15 min at 4°C. The heart, liver, kidney, spleen, testes, and epididymis with the perirenal fat, epididymal fat, and brown fat were harvested. Afterward, each organ was rinsed with physiological saline, weighed, and stored at −70°C for later use.

### 2.6. Histopathological Analysis

The liver tissues were fixed with 10% neutral formalin. Tissue sections were prepared by routine paraffin embedding; 5 *µ*m-thick tissue slides were stained with hematoxylin and eosin (H&E). A veterinary pathologist examined the slides for histopathologic lesions.

### 2.7. Blood Chemistry Analysis

The levels of serum total cholesterol (TC), triglycerides (TGs), and high-density lipoprotein (HDL)-cholesterol were determined with commercial enzymatic kits coupled with a SPOTCHEMTM EZ SP-4430 automated analyzer (ARKRAY, Inc., Kyoto, Japan). Low-density lipoprotein (LDL)-cholesterol levels were calculated using the Friedewald formula (TC-HDL-TG/5). The AI [Atherogenic Index = (TC − HDL-cholesterol)/HDL-cholesterol] and CRF (cardiac risk factor = TC/HDL-cholesterol) were calculated using total serum cholesterol and HDL-cholesterol content [20]. Serum insulin content was analyzed using a mouse insulin ELISA kit (Cusabio Biotech CO., Ltd, USA). Alanine aminotransferase (ALT) levels were also analyzed using an enzymatic colorimetric test. The homeostasis model assessment of insulin resistance (HOMA-IR) was calculated based on fasting insulin levels and fasting glucose levels [21]. HOMA-IR was calculated using the following formula:

### 2.8. Western Blot

Liver tissue (80 mg) was homogenized in RIPA buffer (800 *µ*l) (Thermo Scientific, Rockford, IL). After centrifugation at 14,000 rpm for 15 min at 7°C, the supernatant was used to quantify PPAR-*α*, PPAR-*γ*, TNF-*α*, and GLUT4 protein expression levels via western blot analysis. The total protein content of the supernatant was determined with the BCA reagent method (Thermo scientific, Bartlesville, USA). Equal amounts of protein (30 *µ*g) were separated by 10% sodium dodecyl sulfate polyacrylamide gel electrophoresis and transferred to a nitrocellulose membrane (Hybond, GE Healthcare Life Science, Little Chalfont, UK). The membrane was blocked for 2 h at room temperature with 5% skimmed milk. After overnight incubation at 4°C with either primary anti-mouse PPAR-*α* (Santa Cruz. USA), anti-rabbit PPAR-*γ* (Cell Signaling Technology, Inc.), anti-rabbit TNF-*α* (Cell Signaling Technology, Inc.), or glucose transporter type 4 (GLUT4) (Cell Signaling Technology, Inc.) antibodies, the membrane was washed with TBST and incubated with a horseradish peroxidase-conjugated secondary goat anti-rabbit IgG antibody and a horse anti-mouse IgG (Cell Signaling Technology, Inc.) antibody for 1.5 h at room temperature. Immunodetection was carried out using an ECL detection reagent (SuperSignal, Thermo Scientific, Rockford, IL., USA). All figures showing the results of quantitative analyses (ImageJ, National Institute of Health) include data from at least three independent experiments.

### 2.9. Statistical Analysis

SPSS version 25 (IBM Corp., Armonk, NY, USA) was used for statistical analysis, and all experimental data are presented as the mean ± standard error (SE). One-way ANOVA was performed to test whether the means were significantly different among the groups. Duncan's multiple range analysis was used as a post hoc test to analyze the differences between group pairs. A *p* value <0.05 was considered statistically significant.

## 3. Results

### 3.1. Final Body Weight, Weight Gain, Food Intake, and Food Efficiency Ratio

The final body weight, weight gain, food intake, and food efficiency ratio (FER) are summarized in Table 2. The final body weight was higher in the HFD groups than in the CON group (*p* < 0.05), whereas, in the HFD groups, the final body weight of the HFD + PO10% group was 13% lower than that in the HFD group (*p* < 0.05). There was no significant difference between the HFD group and the HFD + PO5% group. Weight gain was significantly higher in the HFD groups than in the CON group. When comparing weight gains exclusively between the HFD groups, the weight gain in the HFD + PO10% group was 34% lower than that in the HFD group (*p* < 0.05). Food intake was higher in the CON, HFD + PO5%, and HFD + PO10% groups than in the HFD group (*p* < 0.05), with no significant differences between the three groups. The FER was significantly higher in the HFD groups than in the CON group; however, the FER of the HFD + PO10% group was lower than those of both the HFD and HFD + PO5% groups. Energy intake was higher in the HFD groups than in the CON group (*p* < 0.05), but there were no significant differences among the HFD groups.

### 3.2. Organ Weight and Unit Organ Weight

Table 3 shows the total weight and unit total weight of the major organs and body fat for each group. The liver weight in the HFD group was significantly greater than that in the CON group, whereas that in the HFD + PO10% group was lower than that in the HFD group (*p* < 0.05). The liver weight in the HFD + PO5% group was not significantly different from that in the HFD group when comparing only the HFD groups. Furthermore, the heart, kidney, and spleen weights in the HFD groups were significantly greater than those in the CON group, but there were no significant differences among the HFD groups themselves. No differences were identified between the testicular and epididymal weights among any of the experimental groups.

However, the perirenal fat weight was greater in the HFD and HFD + PO5% groups than in the CON group, whereas that of the HFD + PO10% group was significantly less than that of the HFD group (6.3-fold). The epididymal fat weight was significantly less in the CON and HFD + PO10% groups than in the HFD and HFD + PO5% groups (1.5-fold). The brown fat weight was greater in the HFD + PO5% group than in the CON and HFD + PO10% groups but was not different from that in the HFD group.

Regarding the total unit organ weight, although the liver unit organ weight was greater in the CON group than in the HFD groups (*p* < 0.05), there were no significant differences among the HFD groups. The heart and spleen total unit organ weights were also not significantly different among groups. However, the kidney unit organ weight was greater in the CON group than in the HFD and HFD + PO5% groups (*p* < 0.05) but not significantly different from that in the HFD + PO10% group. The testicular unit organ weight was significantly greater in the CON group than in the HFD and HFD + PO5% groups but was not significantly different from that in the HFD + PO10% group. Additionally, no differences between the epididymal unit organ weights were identified among the groups (*p* < 0.05).

Furthermore, the perirenal fat weight was found to be significantly less in the CON group than in the HFD groups. Upon comparing only the HFD groups, the perirenal fat unit weight in the HFD + PO10% group was found to be 1.4-fold less than that in the HFD group; however, there was no significant difference between the weights in the HFD and HFD + PO5% groups. Regarding the epididymal fat unit weight, the HFD + PO10% group tended to have lower values than the HFD and the HFD + PO5% groups; however, the difference between the groups was not significant. There were no differences in the brown fat unit weights in the CON group and the HFD and HFD + PO5% groups, but that in the HFD + PO10% group was less than that in the CON group.

### 3.3. Blood Chemical Analysis

Table 4 shows the effects of *P. oleracea* powder on the blood lipid profiles, insulin levels, HOMA-IR values, and liver function tests of obese mice. The TC level was lower in the CON group than in the HFD groups, albeit not significantly. However, the HDL-cholesterol level in the HFD groups was significantly higher than that in the CON group, and the levels in the HFD + PO5% and HFD + PO10% groups were higher (1.3- and 1.2-fold, respectively) than that the HFD group (*p* < 0.05). The LDL-cholesterol level was lower in the CON group than in the HFD groups, and the level in the HFD + PO10% group was 16.2% lower than that in the HFD group; however, this value was not significantly different from that in the HFD + PO5% group. The TG level was lower in the CON group than in the HFD and HFD + PO10% groups, but there was no significant difference between the HFD group and the HFD + PO5% group. In contrast, the TG levels in the HFD + PO5% and HFD + PO10% groups were lower than those in the HFD group (*p* < 0.05). The TG/HDL-cholesterol index was significantly lower in the HFD + PO5% and HFD + PO10% groups than in the CON and HFD groups. Additionally, the CON, HFD + PO5%, and HFD + PO10% groups exhibited lower AI and CRF values than those in the HFD group, but no significant differences were identified among the three aforementioned groups.

The serum insulin level was significantly higher in the HFD and HFD + PO5% groups than in the CON group, whereas there were no differences between the CON and HFD + PO10% groups. Likewise, the HOMA-IR value, an insulin resistance index, was higher in the HFD and HFD + PO5% groups than in the CON group. On the other hand, the HOMA-IR value in the HFD + PO10% group was lower than that in the HFD group (*p* < 0.05), but there were no differences between the CON and HFD + PO10% groups. ALT, a liver injury index, was the highest in the HFD group (*p* < 0.05), with no significant differences among the remaining three groups (CON, HFD + PO5%, and HFD + PO10%).

### 3.4. Random Blood Glucose Measurement

**Figure 1:**
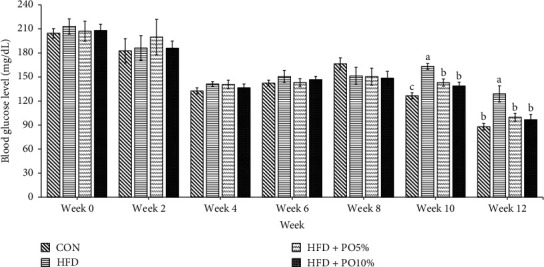
Effect of *P. oleracea* powder on random blood glucose levels at 0, 2, 4, 6, 8, 10, and 12 weeks in mice fed normal diet (CON), high fat diet (HFD), high fat diet with 5% *P. oleracea* powder (HFD+PO5%), and high fat diet with 10% *P. oleracea* powder (HFD+PO10%). Values are the mean ± SE (*n* = 8/group). The same letters represent no significant differences according to the Duncan's multiple range test. *p* < 0.05 was considered significant.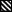 CON: normal diet; 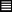 HFD: high fat diet; 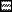 HFD + PO5%: high fat diet with 5% *P. oleracea* powder; 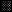 HFD + PO10%: high fat diet with 10% *P. oleracea* powder.

Figure 1 shows the random blood glucose measurements for experimental weeks 0, 2, 4, 6, 8, 10, and 12 (all measurements were obtained at the same time of day). There were no significant differences among groups on weeks 0, 2, 4, 6, and 8. However, the random blood glucose level in week 10 was lower in the CON group than in the HFD groups. When comparing only the three HFD groups, the HFD + PO5% and the HFD + PO10% groups exhibited lower blood glucose levels than the HFD group. The random blood glucose levels in week 12 were found to be similar to those in week 10. However, not only was the random blood glucose level found to be lower in the CON, HFD + PO5%, and HFD + PO10% groups than in the HFD group, but lower glucose levels were also recorded compared to week 10.

### 3.5. Intraperitoneal Glucose Tolerance Test

**Figure 2:**
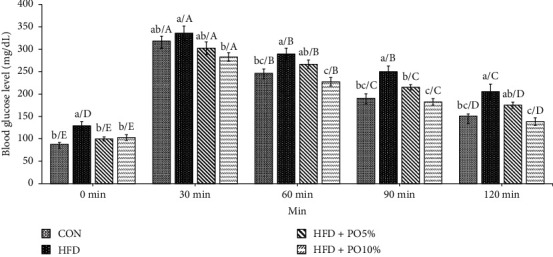
Effect of *P. oleracea* powder, normal diet, and high fat diet on IPGTT in C57BL/6 mice. Values are the mean ± SE (*n* = 8/group). Different lowercase letters show significant differences between groups by Duncan's multiple range test (*p* < 0.05). Different capital letters show significant differences within groups by Duncan's multiple range test (*p* < 0.05). 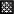 CON: normal diet; 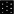 HFD: high fat diet; 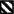 HFD + PO5%: high fat diet with 5% *P. oleracea* powder; 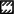 HFD + PO10%: high fat diet with 10% *P. oleracea* powder.

Figure 2 shows the results of the intraperitoneal glucose tolerance test (IPGTT). At the beginning of each experiment, the blood glucose level in each group ranged from 88–129 mg/dl at minute 0. After 30 min, blood glucose levels increased rapidly by a factor of 2.6 to 3.6 compared to the initial blood glucose concentrations and also tended to decrease from 60 to 120 min. After 120 min, when the experiment was concluded, the blood glucose level in the HFD + PO10% group (138.5 mg/dL) was found to be the lowest (*p* < 0.05); however, the difference between the value in this group and that in the CON group (150.9 mg/dL) was not deemed significant. The value in the HFD group was found to be the highest among the experimental groups. When comparing the changes in blood glucose levels at 120 min versus 30 min for each experimental group, the level in the HFD group decreased by 39%, and the levels in the CON group, HFD + PO5% group, and HFD + PO10 group decreased by 53%, 43%, and 51%, respectively.

### 3.6. Effect of *P. oleracea* Powder on Liver Morphology

**Figure 3:**
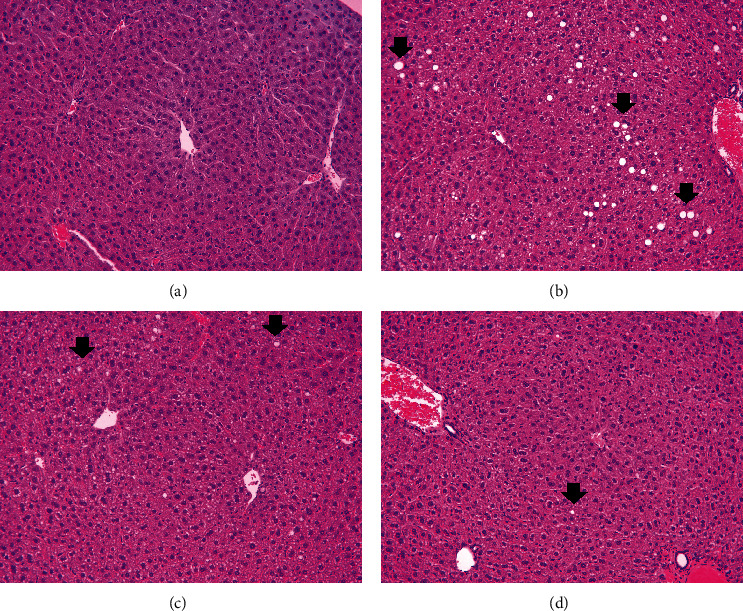
Histopathological analysis of liver in mice administered with high fat diet with low- or high-dose *P. oleracea* powder (5% and 10%, respectively). Arrows indicate the fatty degeneration of hepatocytes. (a) Negative control, (b) high fat diet (HFD), (c) high fat diet with 5% *P. oleracea* powder (HFD+PO5%), and (d) high fat diet with 10% *P. oleracea* powder (HFD+PO10%) (hematoxylin and eosin stain, x200 magnification).

The effects of *P. oleracea* powder on lipid accumulation and cellular swelling in the liver were determined by H&E staining. Considerable lipid accumulation was observed in the livers of HFD-fed mice, as determined by H&E staining (Figure 3(b)). In contrast, *P. oleracea* powder alleviated lipid accumulation to the extent that the HFD + PO10% group showed similar staining to that of the CON group (Figures 3(a), 3(c), and 3(d)). Furthermore, morphological analyses indicated that the lipid droplet size was the largest in the HFD group, followed by the HFD + PO5% group, the HFD + PO10% group, and the CON group (Figure 3). Histopathological lesions of the liver were not observed in the CON group. However, moderate centrilobular ballooning degeneration of the hepatocytes was observed in the HFD, HFD + PO5%, and HFD + PO10% groups (88%, 25%, and 25%, respectively). These results suggest that the administration of *P. oleracea* powder suppressed liver lipid accumulation and hepatocyte cellular swelling.

### 3.7. Effects of *P. oleracea* Powder on the Expression of TNF-*α* Related to Inflammation

**Figure 4:**
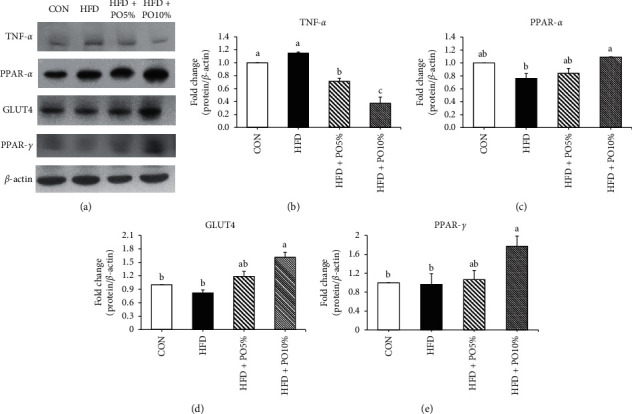
Effect of *P. oleracea* powder on relative protein expression in liver of C57BL/6 mice. Values are the mean ± SE (*n* = 8/group). Values with different letters are significantly different at *p* < 0.05 by Duncan's multiple range test. CON, normal diet; HFD, high fat diet; HFD+PO5%, high fat diet with 5% *P. oleracea* powder; HFD+PO10%, high fat diet with 10% *P. oleracea* powder. (a) Representative western blot bands of TNF-*α*, PPAR-*α*, GLUT4, PPAR-*γ*, and *β*-actin. (b) Relative expression of TNF-*α*/*β*-actin. (c) Relative expression of PPAR-*α*/*β*-actin. (d) Relative expression of GLUT4/*β*-actin. (e) Relative expression of PPAR-*γ*/*β*-actin.

The protein expression level of TNF-*α* in liver tissue was measured via Western blot (Figures 4(a) and 4(b)). There were no observable differences between the CON and HFD groups, whereas the HFD + PO5% and HFD + PO10% groups displayed decreased protein expression levels of TNF-*α* compared with those in the CON group (*p* < 0.05). When comparing the HFD groups only, the expression levels in the HFD + PO5% and HFD + PO10% groups were significantly lower than those in the HFD group.

### 3.8. Effects of *P. oleracea* Powder on Fatty Acid Oxidation in the Liver

The expression of PPAR-*α*, a protein that controls fatty acid oxidation, was measured in liver tissue (Figures 4(a) and 4(c)). No between-group differences were observed among the CON, HFD + PO5%, and HFD groups; however, the expression levels of PPAR-*α* in the HFD + PO10% group were higher than those in the HFD group (*p* < 0.05).

### 3.9. Effects of *P. oleracea* Powder on Insulin Resistance in the Liver

The protein expression levels of GLUT4 and PPAR-*γ* were analyzed as indicators of insulin resistance (Figures 4(a), 4(d), and 4(e)). The GLUT4 expression level found in the HFD + PO10% group was significantly higher than those in the CON, HFD, and HFD + PO5% groups; however, no significant difference was observed among the latter three groups. Likewise, the protein expression level of PPAR-*γ* was also found to be significantly higher in the HFD + PO10% group than in the CON, HFD, and HFD + PO5% groups; furthermore, there were no significant differences among the CON, HFD, and HFD + PO5% groups.

## 4. Discussion

The diet-induced obese mice used in this study constitute an important model to elucidate the correlation between several chronic diseases and fat [22]. In a previous study, an HFD group fed a *P. oleracea* ethanol (PE) extract mix exhibited significantly decreased body weights compared with an HFD-fed group that did not receive PE [17]. In addition, Won and Kim [18] reported that the FER was significantly lower in mice treated with high-dose ethanol extracts from *P. oleracea* than in the HFD group. These observations were consistent with our results; they demonstrate that *P. oleracea* powder reduces body weight by decreasing FER and suppressing body weight gain. Although there were differences in diet calories by adding *P. oleracea* powder (5% and 10%) in the HFD diet proportionally, the result of energy intake (kcal/day) showed no differences among the HFD groups (*p* < 0.05). That is, the overall efficacy of *P. oleracea* powder was due to *P. oleracea* powder itself rather than lesser diet calories.

In another study, epididymal fat was significantly reduced in stevia-treated groups compared with an HFD control (HC) group [23]. Additionally, the epididymal fat weight was significantly reduced in the doenjang-supplemented HFD group compared with the HFD group [24]. These observations were consistent with our results. PPAR-*α* is abundantly present in brown fat and the liver and is known to inhibit the growth and differentiation of adipocytes, promote lipolysis, and regulate lipoprotein synthesis and tissue inflammatory responses. This protein is also known to stimulate carnitine palmitoyltransferase I (CPT-1) to promote the *β*-oxidation of FFAs [8]. In this study, the HFD + PO10% group exhibited the highest PPAR-*α* protein expression level. Administration of *P. oleracea* powder is thought to inhibit fat accumulation by promoting fatty acid oxidation in the body through increased PPAR-*α* expression.

However, brown fat, which is characterized by energy consumption and heat generation, is known to play an important role in adaptive thermogenesis to allow for the maintenance of heat balance and basic body temperature [25, 26]. This role of brown fat is known to be regulated by uncoupling proteins (UCPs) present in the inner mitochondrial membrane [26]. Previous studies have reported that the weight of brown fat per body weight unit is significantly higher in HFD groups than in normal chow groups and treated groups [27, 28]. Although the fat pad weight of brown adipose tissue in the royal jelly (RJ) group was lower, it was reported that the RJ group had higher levels of UCP-1 and cytochrome c oxidase subunit IV (COX-IV) protein expression, thereby increasing thermogenesis for energy consumption [28]. In this study, the brown fat weight in the HFD groups was also higher than that of the CON group. There was no difference in the weight of brown fat between the HFD + PO10% and HFD groups; however, based on previous studies' results, body weight is likely controlled through thermogenesis for energy consumption via the expression of both UCP-1 and COX-IV. Additional studies are required to confirm this hypothesis.

Obesity reportedly increases blood levels of TC, LDL-cholesterol, VLDL-cholesterol, and TG while also decreasing HDL-cholesterol levels. This increases the likelihood that an obese subject will experience cardiovascular diseases such as hyperlipidemia, hypercholesterolemia, and atherosclerosis [2, 29]. Additionally, chronically excessive fat intake is known to affect vascular health through oxidative and inflammatory reactions caused by metabolic stress [30]. In a previous study, HDL-cholesterol levels were higher in garlic shoot extract-treated groups than in a control group, whereas LDL-cholesterol and VLDL-cholesterol levels were lower, and the AI and CRF were significantly lower [31]. The AI is known as a risk indicator for coronary artery disease and CRF is known as a hyperlipidemia risk indicator [32]. In our study, the HDL-cholesterol, AI, and CRF values in the HFD + PO5% and the HFD + PO10% groups were significantly lower than those in the HFD group; therefore, administration of *P. oleracea* powder may be an effective measure to prevent cardiovascular disease.

Furthermore, TNF-*α*, a proinflammatory cytokine, is known to regulate leukocyte adhesion and migration in vascular inflammatory diseases including atherosclerosis [33]. In our study, *P. oleracea* powder administration resulted in significantly lower TNF-*α* protein expression in the *P. oleracea*-treated groups than in the HFD group (Figures 4(a) and 4(b)). According to a study, an aqueous extract of *P. oleracea* reportedly prevented vascular inflammatory processes not only by inhibiting intracellular reactive oxygen species (ROS) production and nuclear factor (NF)-*κ*B activation but also by reducing the overexpression of vascular cell adhesion molecule 1 (VCAM-1), intercellular adhesion molecule 1 (ICAM-1), and endothelial cell selectin (E-selectin) [34]. Therefore, the administration of *P. oleracea* powder both improves blood lipid fractions and cardiovascular diseases such as atherosclerosis.

Additionally, an HFD is known to induce hyperinsulinemia and hyperglycemia [3], and increased serum insulin levels and HOMA-IR values are indicative of insulin resistance [35]. In this study, blood glucose levels at week 10 and week 12 were lower in the *P. oleracea*-treated group than in the HFD group (*p* < 0.05). The HOMA-IR and IPGTT values were lower in the HFD + PO10% group than in the HFD group (*p* < 0.05).

A previous study involved 4-week-old C57BL/6 mice being orally administered with 0%, 0.2%, 0.5%, and 1%, respectively, of Artemisia Princeps ethanol (APE) extract once a day for 14 weeks [36]. In the normal diet and high-fat diet, the blood glucose level in the oral glucose tolerance test (OGTT) was lower in the HF-1.0%APE group than in the HF-0%APE group (*p* < 0.05), plasma insulin level was not a significant difference between the HF-0.5%APE group and HF-0%APE group, but the HF-0.5%APE group was lower than the HF-0%APE group in HOMA-IR (*p* < 0.05) [37]. These results show that glucose tolerance can be improved by improving the HOMA-IR value, which is a good predictor of insulin sensitivity [37]. In a previous study, an HFD group exhibited the highest HOMA-IR value (*p* < 0.05), whereas the values in mulberry anthocyanin groups were significantly higher than those in a low-fat diet (LFD) group [35]. These observations are consistent with our results. Therefore, *P. oleracea* may reduce HOMA-IR values, which in turn relieves type 2 diabetes by decreasing insulin resistance.

Adiponectin is an adipokine that is exclusively secreted from adipose tissue into the bloodstream and accounts for 0.01% of total plasma protein. Oral administration of adiponectin to obese and diabetic mice results in decreases in both body weight and blood glucose levels and enhanced insulin sensitivity [9]. TNF-*α* secretion increases dramatically in both obese subjects and patients with diabetes, and it directly contributes to the reduction of adiponectin [10]. Sahin et al. [10] demonstrated that the expression levels of PPAR-*γ* in treated groups were significantly higher than those in an HFD/streptozotocin (STZ) group. Additionally, the expression of PPAR-*γ* and adiponectin was higher in the treated group than in the HFD group (*p* < 0.05), whereas the expression of TNF-*α* was significantly lower [23]. Our results in this study were consistent with those of the abovementioned study.

An increase in GLUT4 expression is known to reduce insulin resistance [38]. Glucose utilization decreases in insulin-resistant muscle, and the expression of GLUT4 protein and mRNA in skeletal muscle decreases with an HFD [37]. In previous studies, GLUT4 and PPAR-*γ* protein expressions were significantly increased in treated groups compared with a diabetic control group [39, 40]; these observations were consistent with our results.

## 5. Conclusion

In conclusion, our findings indicate that body weight gain and perirenal and epididymal fat tissues were reduced significantly in the high *P. oleracea* powder group along with increased HDL-cholesterol and decreased HOMA-IR. PPAR-*α*, GLUT4, and PPAR-*γ* protein expression were upregulated in the high *P. oleracea* powder group compared to the HFD group, while TNF-*α* protein expression was downregulated in the *P. oleracea*-treated groups than in the HFD group. We concluded that the *P. oleracea* powder was effective at suppressing body weight gain, reducing body fat, regulating blood glucose level and insulin sensitivity, and decreasing inflammation.

## Figures and Tables

**Table 1:** Composition of diet formula (%).

Ingredient	CON^(1)^	Ingredient	HFD^(2)^	HFD + PO5%	HFD + PO10%
Ground whole hard meal	34.90	Casein	26.50	25.18	23.85
Ground ^#^2 yellow corn	21.00	L-cysteine	0.40	0.38	0.36
Ground whole oats	10.00	Maltodextrin	16.00	15.2	14.4
Wheat middlings	10.00	Sucrose	9.00	8.55	8.10
Fish meal (60% protein)	9.00	Lard	31.00	29.45	27.9
Soy oil	2.00	Soybean oil	3.00	2.85	2.7
Soybean meal (47.5% protein)	5.00	Cellulose	6.55	6.55	5.90
Alfalfa meal (17% protein)	2.00	Mineral mix^(3)^	4.80	4.56	4.32
Corn gluten meal (60% protein)	2.00	Calcium phosphate	0.34	0.32	0.31
Dicalcium phosphate	1.50	Vitamin mix^(4)^	2.10	2.0	1.89
Yeast-brewer	1.00	Choline bitartrate	0.30	0.29	0.27
Premixes	0.60	Blue food color	0.01	0.01	0.01
Ground limestone	0.50	*Portulaca oleracea* powder^(5)^	—	5.00	10.00
Salt	0.50	Energy (kcal/g) ^(6)^	5.10	4.86	4.61
Energy (kcal/g)	3.11	—			

^(1)^CON: control diet; HFD: high fat diet; HFD + PO5%: high fat diet + 5% *P. oleracea* powder; HFD + PO10%: high fat diet + 10% *P. oleracea* powder. ^(2)^HFD was composed of 60% kcal from fat (protein 23.5%, carbohydrate 27.3%, and fat 34.3% by weight). ^(3)^Mineral mixture according to AIN-93G-MX. ^(4)^Vitamin mixture according to AIN-93-VX. ^(5)^The powder was purchased from Hanbit Farm (Yeongdeok, Kyeongbuk, Korea) and composed of protein 13.4%, fat 1.9%, nitrogen-free extract 47.6%, ash 12.1%, and fiber 25.0% based on Standard Tables of Feed Composition from Korea National Institute of Animal Science. ^(6)^Calculated by *Portulaca oleracea* nutrient based on Food Data Central of U.S Department of Agriculture.

**Table 2:** Effect of experimental diets on body weight gain and daily food intake.

Item	Group^(1)^			
	CON	HFD	HFD + PO5%	HFD + PO10%
Initial body weight (g)	21.7 ± 0.30^(3)NS^	20.7 ± 0.45	20.9 ± 0.49	21.8 ± 0.17
Final body weight (g)	27.9 ± 0.31^c^	39.3 ± 1.67^a^	37.8 ± 1.30^ab^	34.2 ± 1.36^b^
Body weight gain (g/12 weeks)	6.30 ± 0.39^c^	18.6 ± 1.62^a^	16.9 ± 1.18^a^	12.3 ± 1.32^b^
Daily food intake (g/day)	2.82 ± 0.07^b^	2.45 ± 0.07^a^	2.62 ± 0.03^b^	2.71 ± 0.03^b^
FER^(2)^	0.027 ± 0.002^c^	0.091 ± 0.007^a^	0.078 ± 0.006^a^	0.054 ± 0.006^b^
Energy intake (kcal/day)	8.79 ± 0.22^b^	12.5 ± 0.34^a^	12.7 ± 0.17^a^	12.5 ± 0.13^a^

^(1)^CON: control; HFD: high fat diet; HFD + PO5%: high fat diet + 5% *P. oleracea* powder; HFD + PO10%: high fat diet + 10% *P. oleracea* powder. ^(2)^FER (food efficiency ratio) = body weight gain/food intake per week. ^(3)^Values are the mean ± SE (*n* = 8/group). ^(a–c)^Values with a different superscript in the same row are significantly different at *p* < 0.05 by Duncan's multiple range test. NS: not significant.

**Table 3:** Organ weight and unit organ weight of C57BL/6 mice fed the experimental diets.

Group^(1)^	Total organ weight (g)				Total unit organ weight (mg/g BW)			
	CON	HFD	HFD + PO5%	HFD + PO10%	CON	HFD	HFD + PO5\%	HFD + PO10%
Liver	0.99 ± 0.012^(2)b^	1.08 ± 0.046^a^	1.03 ± 0.024^ab^	0.98 ± 0.018^b^	35.35 ± 0.331^a^	27.43 ± 0.598^b^	27.50 ± 0.712^b^	29.06 ± 1.124^b^
Heart	0.10 ± 0.005^b^	0.13 ± 0.006^a^	0.13 ± 0.006^a^	0.12 ± 0.003^a^	3.64 ± 0.197^NS^	3.40 ± 0.176	3.56 ± 0.157	3.63 ± 0.199
Kidney	0.33 ± 0.004^b^	0.37 ± 0.008^a^	0.37 ± 0.009^a^	0.36 ± 0.013^a^	11.79 ± 0.182^a^	9.39 ± 0.363^c^	9.91 ± 0.376^bc^	10.70 ± 0.535^ab^
Spleen	0.05 ± 0.003^b^	0.06 ± 0.003^a^	0.06 ± 0.003^a^	0.06 ± 0.002^a^	1.61 ± 0.089^NS^	1.61 ± 0093	1.59 ± 0.049	1.74 ± 0.104
Testis	0.19 ± 0.013^NS^	0.19 ± 0.011	0.18 ± 0.015	0.18 ± 0.008	6.66 ± 0.523^a^	4.84 ± 0.413^b^	4.82 ± 0.471^b^	5.34 ± 0.423^ab^
Epididymidis	0.02 ± 0.002^NS^	0.02 ± 0.002	0.02 ± 0.002	0.02 ± 0.002	0.58 ± 0.065^NS^	0.49 ± 0.065	0.47 ± 0.047	0.52 ± 0.057
Perirenal fats	0.51 ± 0.055^c^	2.15 ± 0.177^a^	1.95 ± 0.093^a^	0.34 ± 0.146^b^	18.23 ± 1.878^c^	54.42 ± 3.006^a^	51.79 ± 2.673^a^	38.89 ± 4.002^b^
Epididymal fats tissue	0.02 ± 0.002^b^	0.03 ± 0.003^a^	0.03 ± 0.002^a^	0.02 ± 0.003^b^	0.63 ± 0.061^NS^	0.77 ± 0.070	0.77 ± 0.065	0.63 ± 0.089
Brown fats tissue	0.08 ± 0.004^bc^	0.10 ± 0.011^ab^	0.10 ± 0.008^a^	0.07 ± 0.005^c^	2.69 ± 0.137^a^	2.45 ± 0.240^ab^	2.69 ± 0.209^a^	2.04 ± 0.161^b^

^(1)^CON: control diet; HFD: high fat diet; HFD + PO5%: high fat diet + 5% *P. oleracea* powder; HFD + PO10%: high fat diet + 10% *P. oleracea* powder. ^(2)^Values are the mean ± SE (*n* = 8/group). ^(a–c)^Values with different superscript in the same row are significantly different at *p* < 0.05 by Duncan's multiple range test. NS: not significant.

**Table 4:** Blood serum lipid parameter of C57BL/6 mice fed the experimental diets.

Item	Group^(1)^			
	CON	HFD	HFD + PO5%	HFD + PO10%
TC (mg/dL)	59.2 ± 3.4^(6)b^	81.8 ± 6.4^a^	89.5 ± 5.5^a^	92.6 ± 0.5^a^
HDL-cholesterol (mg/dL)	44.3 ± 3.0^c^	58.3 ± 5.8^b^	75.0 ± 4.9^a^	72.5 ± 0.2^a^
LDL-cholesterol (mg/dL)	6.3 ± 0.3^c^	11.7 ± 0.4^a^	11.3 ± 0.8^ab^	9.8 ± 0.7^b^
Triglyceride (mg/dL)	43.5 ± 1.0^c^	59.5 ± 1.4^a^	46.0 ± 3.7^bc^	51.5 ± 1.9^b^
TG/HDL-cholesterol	1.01 ± 0.05^a^	1.08 ± 0.08^a^	0.64 ± 0.07^b^	0.71 ± 0.03^b^
AI^(2)^	0.34 ± 0.01^b^	0.43 ± 0.03^a^	0.29 ± 0.03^b^	0.28 ± 0.01^b^
CRF^(3)^	1.34 ± 0.01^b^	1.43 ± 0.03^a^	1.29 ± 0.03^b^	1.28 ± 0.01^b^
Insulin (*μ*U/ml)	18.8 ± 2.85^b^	39.3 ± 4.92^a^	39.4 ± 2.68^a^	27.5 ± 5.23^ab^
HOMA-IR^(4)^	4.2 ± 0.7^b^	10.6 ± 2.2^a^	7.6 ± 1.4^ab^	5.2 ± 1.5^b^
ALT (U/L)^(5)^	53.0 ± 8.7^b^	135.5 ± 14.0^a^	46.8 ± 3.5^b^	40.3 ± 4.5^b^

^(1)^CON: control diet; HFD: high fat diet; HFD + PO5%: high fat diet + 5% *P. oleracea* powder; HFD + PO10%: high fat diet + 10% *P. oleracea* powder. ^(2)^AI (atherogenic index) = total cholesterol-HDL-cholesterol/HDL-cholesterol. ^(3)^CRF (cardiac risk factor) = total cholesterol/HDL cholesterol. ^(4)^HOMA-IR (homeostasis model assessment of insulin resistance) = fasting insulin level (*μ*U/mL)^*∗*^fasting glucose level (mg/dL)/405. ^(5)^Alanine aminotransferase.^(6)^Values are the mean ± SE (*n* = 8/group). ^(a–c)^Values with a different superscript in the same row are significantly different at *p* < 0.05 by Duncan's multiple range test.

## Data Availability

The data used to support the findings of this study are available from the corresponding author upon request.
